# Delay of the Onset of Puberty in Female Rats by Prepubertal Exposure to T-2 Toxin

**DOI:** 10.3390/toxins7114668

**Published:** 2015-11-09

**Authors:** Rong Yang, Yi-Mei Wang, Li-Shi Zhang, Li Zhang, Zeng-Ming Zhao, Jun Zhao, Shuang-Qing Peng

**Affiliations:** 1 Evaluation and Research Center for Toxicology, Institute of Disease Control and Prevention, Academy of Military Medical Sciences, Beijing 100071, China; E-Mails: yangrongscstu@126.com (R.Y.); zhangli_526@163.com (L.Z.); zhaozm000@163.com (Z.-M.Z.); zj35566@sina.com (J.Z.); 2 West China School of Public Health, Sichuan University, Chengdu 610041, China; E-Mail: lishizhang_56@163.com

**Keywords:** T-2 toxin, female rat, puberty onset, delay, HPG axis, GnRH

## Abstract

Growing evidence has revealed the deleterious influence of environmental and food contaminants on puberty onset and development in both animals and children, provoking an increasing health concern. T-2 toxin, a naturally-produced Type A trichothecene mycotoxin which is frequently found in cereal grains and products intended for human and animal consumption, has been shown to impair the reproduction and development in animals. Nevertheless, whether this trichothecene mycotoxin can disturb the onset of puberty in females remains unclear. To clarify this point, infantile female rats were given a daily intragastric administration of vehicle or 187.5 μg/kg body weight of T-2 toxin for five consecutive days from postnatal day 15 to 19, and the effects on puberty onset were evaluated in the present study. The results revealed that the days of vaginal opening, first dioestrus, and first estrus in regular estrous cycle were delayed following prepubertal exposure to T-2 toxin. The relative weights of reproductive organs uterus, ovaries, and vagina, and the incidence of corpora lutea were all diminished in T-2 toxin-treated rats. Serum levels of gonadotropins luteinizing hormone, follicle-stimulating hormone, and estradiol were also reduced by T-2 toxin treatment. The mRNA expressions of hypothalamic gonadotropin-releasing hormone (GnRH) and pituitary GnRH receptor displayed significant reductions following exposure to T-2 toxin, which were consistent with the changes of serum gonadotropins, delayed reproductive organ development, and delayed vaginal opening. In conclusion, the present study reveals that prepubertal exposure to T-2 toxin delays the onset of puberty in immature female rats, probably by the mechanism of disturbance of hypothalamic-pituitary-gonadal (HPG) axis function. Considering the vulnerability of developmental children to food contaminants and the relative high level of dietary intake of T-2 toxin in children, we think the findings of the present study provide valuable information for the health risk assessment in children.

## 1. Introduction

Puberty, marked by the appearance of secondary sexual characteristics, acceleration of growth, and eventual capacity for fertility, is controlled by the activation of the hypothalamic-pituitary-gonadal (HPG) axis [1,2]. Accordingly, exogenous or endogenous factors that can dysregulate the HPG axis potentially influence the pubertal onset and development, resulting in advanced or delayed puberty. There has been a worldwide scientific discussion on the potential pubertal development consequences of children exposed to various xenobiotics, especially environmental and food contaminants, and there is a growing body of epidemiologic and experimental evidence for the xenobiotics’ influence on puberty onset in both males and females [3,4,5,6,7,8,9].

Fusariotoxins the secondary metabolites of *Fusarium* fungi, such as T-2 toxin, fumonisin B1 and zearalenone known as one kind of the most hazardous food contaminants, are commonly detected from cereals and products intended for human and animal consumption worldwide [10,11]. Available data have indicated the adverse effects of fusariotoxins on reproduction and development in animals [12], thus provoking an increasing health concern. Zearalenone was suspected to be a triggering factor for true precocious puberty development in girls [5], and there was evidence that sexual maturation could be advanced by zearalenone in female mice [13]. Ewuola and Egbunike [14] revealed that dietary fumonisin B1 exposure caused puberty onset delay in male rabbits, and sexual maturity delay in growing male pigs fed dietary fumonisin B1 was also found by Gbore [15]. It has been proved that T-2 toxin, the most toxic Type A trichothecenes can pass through the placenta [16,17], and exerts strong reproductive and developmental toxicities [18,19]. It was revealed that exposure to T-2 toxin reduced steroidogenesis and induced alterations in steroidogenic gene expressions in H295R cells, indicating its potential endocrine disrupting activity [20]. Wu *et al.* [21] recently finds that T-2 toxin regulates steroid hormone secretion of rat ovarian granulosa cells through cAMP-PKA pathway. More recently, Liu *et al.* [22] reveals that low doses of T-2 toxin stimulates GnRH secretion in GT1-7 cells, an immortalized hypothalamic neuron cell line. In view of the crucial role of GnRH secretion in the activation of HPG axis, the findings of Liu *et al.* suggest the potential influence of T-2 toxin on pubertal onset and development.

To the best of our knowledge, no *in vivo* study has described the effect of T-2 toxin on pubertal onset and development. In consideration of T-2 toxin induced reproductive and developmental disorders and its regulatory activity on hypothalamic neuron GnRH secretion mentioned above, we hypothesize reasonably that T-2 toxin may also disturb the onset and development of puberty. The sex development process of rodents and humans are similar, both experiencing similar central activation and endocrine changes, and the established time of puberty in rats is short (2–3 months). Therefore, the present study was undertaken to explore the effect of prepubertal exposure to T-2 toxin on the onset of puberty in immature female rats with an exposure dose related to the dietary exposure in humans. The alterations in HPG axis which determines the onset of puberty were also detected.

## 2. Results

### 2.1. General Observations of Female Rats Following Prepubertal Exposure to T-2 Toxin

No abnormal signs in general appearance and mortality were observed in all the infantile female rats during the experiment. All the rats displayed normal activities and growth, and histological (macroscopic and microscopic) examinations of main tissues/organs, such as thymus, liver, heart, kidney, spleen and bone, showed no any abnormalities or lesions in any rat. No significant difference in body weight was identified between control and T-2 toxin treatment group when compared at the same age (postnatal day 15 (PND15), PND19, and PND35) (Table 1).

**Table 1:** Effect of prepubertal exposure to T-2 toxin on the body weight of female rats.

Groups	Body Weight (g)		
	PND15	PND19	PND35
Vehicle control	37.13 ± 1.18	47.18 ± 2.20	143.56 ± 5.80
T-2 toxin treatment	37.42 ± 1.22	47.73 ± 1.11	142.98 ± 5.26

The data are expressed as means ± SD (*n* = 10).

### 2.2. Effects of Prepubertal Exposure to T-2 Toxin on the Days of Vaginal Opening (VO), First Dioestrus (D1), and First Estrus (E1) in Regular Estrous Cycles

**Figure 1:**
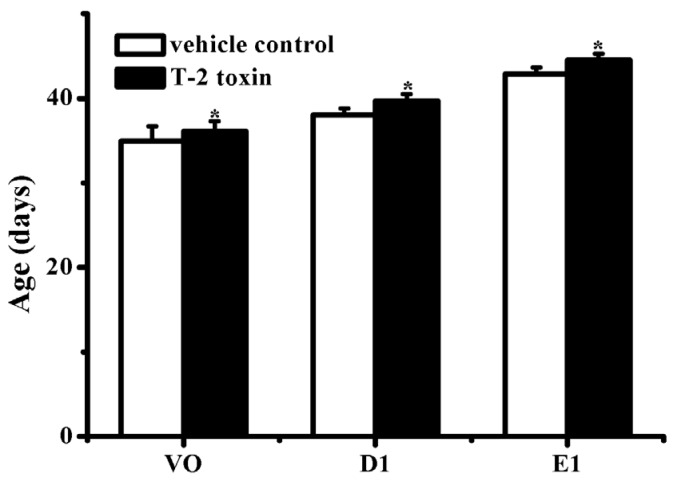
Effects of prepubertal exposure to T-2 toxin on the ages of VO, D1, and E1 of female rats. Data were means ± SD (*n* = 10). VO, vaginal opening; D1, first dioestrus; E1, first estrus. * *p* < 0.05 *versus* vehicle control group.

In female rats, vaginal opening and the emergence of the first diestrus are decisive markers of puberty. As compared with the vehicle control group, the days of VO, D1, and E1 of the female rats in T-2 toxin treatment group were all delayed (*p* < 0.05, *p* < 0.05, *p* < 0.05, respectively) (Figure 1).

### 2.3. Effect of Prepubertal Exposure to T-2 Toxin on the Vaginal Estrus

In the current study, irregular estrous cyclicity at an early age was observed in all female rats. However, the irregularities were transient and succeeded by periods of normal four- and five-day cycles (including esturs, metestrus, diestrus, and proestrus) in both groups. Additionally, the performance of vaginal smears and the cycle length in each group were similar.

### 2.4. Effect of Prepubertal Exposure to T-2 Toxin on the Development of Reproductive Organs

The development of reproductive organs is often used as a reliable indicator of the appearance of secondary sexual characteristics in female rats. As shown in Table 2, the relative weights of uterus and ovaries, and the incidence of corpora lutea were markedly decreased in T-2 toxin treatment group (*p* < 0.05, *p* < 0.05, and *p* < 0.01, respectively) as compared with the control ones. The relative vaginal weight was also reduced despite no significant difference.

**Table 2:** Effects of prepubertal exposure to T-2 toxin on the development of reproductive organs of female rats.

Groups	Relative Organic Weight (mg/g)			Corpora Lutea
	Uterus	Ovary	Vagina	
Vehicle control	1.16 ± 0.28	0.49 ± 0.10	1.05 ± 0.27	9.6 ± 4.7
T-2 toxin treatment	0.83 ± 0.40 *	0.33 ± 0.08 *	0.91 ± 0.31	1.6 ± 0.6 **

Data are presented as the means ± SD of 10 animals. * *p* < 0.05, ** *p* < 0.01 *versus* vehicle control group.

### 2.5. Histopathological Alterations of Reproductive Organs

#### 2.5.1. Uterus

**Figure 2:**
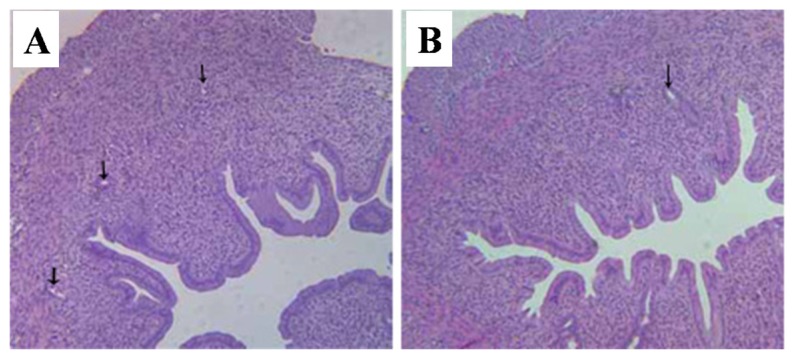
Representative histopathological pictures of uterus stained by H and E. (**A**) vehicle control group; (**B**) T-2 toxin treatment group. The arrows indicate endometrial glands. Magnification ×100.

As shown in Figure 2, the thickness of myometrium and the number of endometrial glands were significantly reduced in T-2 toxin treated rats as compared with the controls.

#### 2.5.2. Ovaries

**Figure 3:**
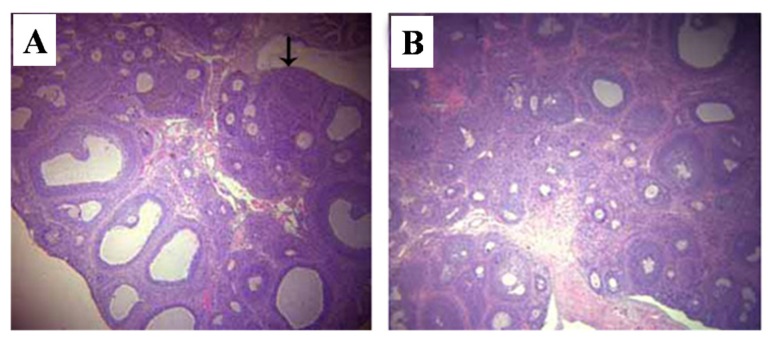
Representative histopathological pictures of ovary stained by H and E. (**A**) vehicle control group; (**B**) T-2 toxin treatment group. The arrow indicates the corpus luteum. Magnification ×40.

In control rats, the major ovarian follicles were secondary follicles, and there was little corpus lutea. In contrast, there were more primary follicles and scanty corpus lutea in T-2 toxin treated rats (Figure 3).

#### 2.5.3. Vagina

**Figure 4:**
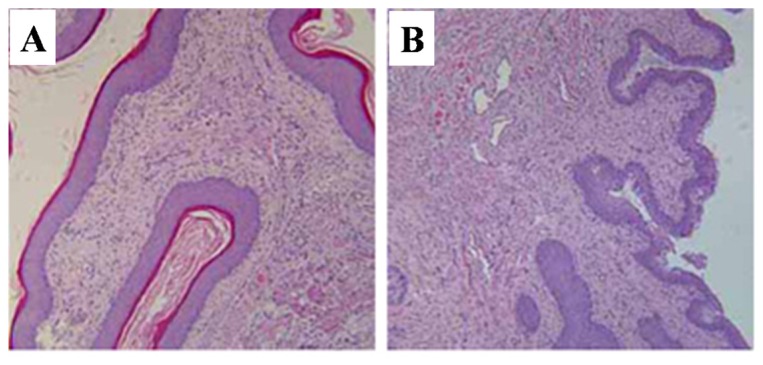
Representative histopathological pictures of vagina stained by H and E. (**A**) vehicle control group; (**B**) T-2 toxin treatment group. Magnification ×100.

When compared with the control rats, the thickness of vaginal wall, the layer of mucosal epithelial and keratinization of the epithelium were all decreased in T-2 toxin treated rats (Figure 4).

### 2.6. Effects of Prepubertal Exposure to T-2 Toxin on the Levels of Serum Gonadotropins

Gonadotropins luteinizing hormone (LH), follicle-stimulating hormone (FSH) and estradiol are responsible for the development of reproductive function and secondary sexual characteristics. The levels of serum gonadotropins are therefore effective targets to determine the onset of maturity. In the present study, serum levels of LH and estradiol were reduced by as much as 20% and 24% (*p* < 0.01, *p* < 0.05) in T-2 toxin treatment group in comparison to vehicle control group, respectively. Serum FSH concentration also presented a similar change despite no significant difference (Table 3).

**Table 3:** Effects of prepubertal exposure to T-2 toxin on serum levels of gonadotropins in female rats.

Groups	Serum Gonadotropins		
	LH (ng/mL)	FSH (ng/mL)	Estradiol (pg/mL)
Vehicle control	8.84 ± 0.39	9.65 ± 0.95	43.42 ± 4.58
T-2 toxin treatment	7.03 ± 0.17 **	8.19 ± 0.78	33.06 ± 3.04 *

Data are presented as the means ± SD of 10 animals. * *p* < 0.05, ** *p* < 0.01 *versus* vehicle control group.

### 2.7. Effects of Prepubertal Exposure to T-2 Toxin on the mRNA Expressions of GnRH and GnRHR

**Figure 5:**
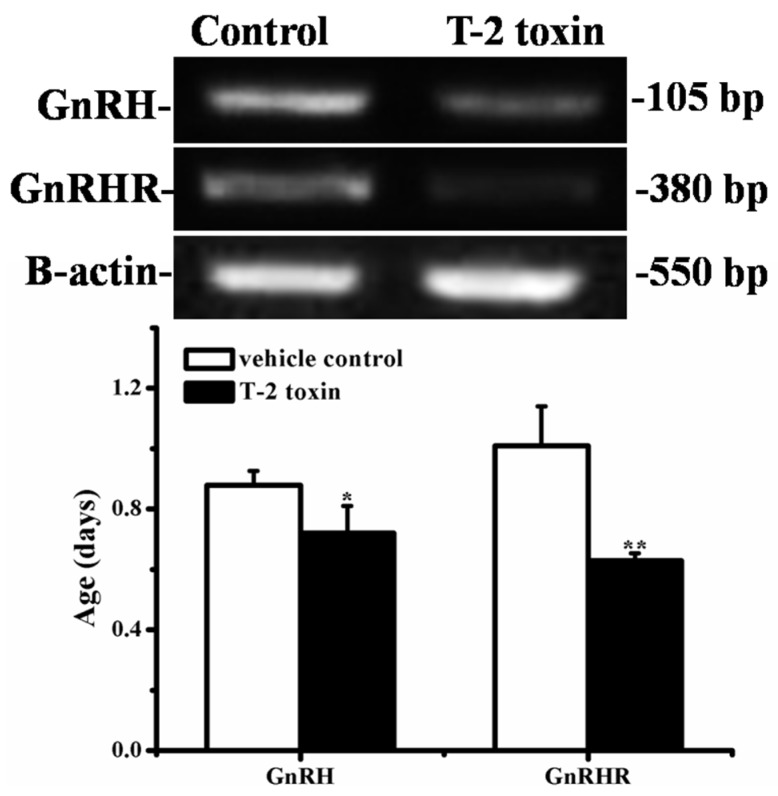
The expressions of hypothalamic GnRH and pituitary GnRHR by RT-PCR (BioRad, Hercules, CA, USA) analysis. The upper picture shows the gel electrophoresis of the RT-PCR products for the hypothalamic GnRH and pituitary GnRHR amplified from the total RNA isolated from the vehicle control and T-2 toxin treatment groups in Experiment II. The results of GnRH and GnRHR were normalized to β-actin level. Data are expressed as the means ± SD of 10 animals. * *p* < 0.05, ** *p* < 0.01 *versus* vehicle control group.

As depicted in Figure 5, hypothalamic GnRH mRNA expression was notably reduced in T-2 toxin treated rats as compared to the controls (*p* < 0.05). A similar decrease in pituitary GnRHR mRNA expression was also observed (*p* < 0.01) following T-2 toxin treatment.

## 3. Discussion

The present findings clearly show that prepubertal exposure to T-2 toxin causes a delay of puberty onset in infantile female rats without showing any visible toxicity. Vaginal opening and the first ovulation are decisive markers of puberty in female rats [23], and the indirect marker of the first ovulation is the emergence of the first diestrus. Age of the first estrus, which occurs the day after the first preovulatory surge of gonadotropins, is an endpoint used to assess the completion of the pubertal process in females [24]. Many studies have used the age of vaginal opening and the emergence of the first diestrus as efficient indicators of puberty in female rats [25,26]. A previous study indicated that the day of vaginal opening of female rats was around the 34th day [27], which is consistent with our current result. In the present study, the days of vaginal opening, first diestrus, and first estrus were all postponed by T-2 toxin, suggesting that prepubertal exposure to T-2 toxin can delay the onset of puberty. The development of reproductive organs is usually adopted as an adequate indicator of the appearance of secondary sexual characteristics in female rats. The present study revealed that T-2 toxin treatment for five consecutive days at a daily dose of 187.5 μg/kg bw diminished the relative weights of uterus, ovaries, and vagina (Table 2), and reduced the incidence of corpora lutea (Table 2). These above results combined with the abnormal histological findings of the reproductive organs (Figure 2, Figure 3 and Figure 4), indicated that prepubertal exposure to T-2 toxin induced a delay of reproductive organ development.

The physical changes associated with puberty are caused by prior activation of hypothalamic-pituitary-gonadal (HPG) axis. The initiation of HPG axis requires an increase in pulsatile release of GnRH from the hypothalamus. Thereafter, GnRH binds with high affinity to its specific receptor GnRHR on the surface of gonadotrope pituitary cells, leading to the synthesis and release of gonadotrophins such as LH and FSH from the anterior pituitary gland [28]. LH and FSH, which in turn stimulates GnRH secretion [29], eliciting the secretions of gonadal hormones estrogen and testosterone, which are responsible for the development of reproductive function and secondary sexual characteristics. Therefore, the levels of serum gonadotrophins are also effective targets to determine the onset of female maturation. In the present study, prepubertal exposure to T-2 toxin reduced the serum levels of FSH, LH and estradiol of female rats (Table 3), indicating that T-2 toxin could delay the onset of maturation. These hormone changes were well consistent with delayed reproductive organ development.

GnRH, the pulsatile release of which initiates HPG axis, is located mainly in the hypothalamus. Studies have proved that the onset of puberty in humans and rodents is highlighted by the augmentation of pulsatile GnRH secretion [30,31]. More recently, our team finds that T-2 toxin can regulate GnRH secretion in hypothalamic GT1-7 cells *in vitro* [22]. In that study, GT1-7 cells were treated with a series of low concentrations of T-2 toxin (0.01, 0.05, 0.1, 0.5 and 1 ng/mL) for 3 h and 6 h, respectively. Moderate increases in GnRH level were observed for 6 h of T-2 toxin treatment at the concentrations of 0.05, 0.1 and 0.5 ng/mL, but not for 3 h of treatment and higher concentration. However, in our present study *in vivo*, prepubertal exposure to T-2 toxin decreased mRNA expressions of hypothalamic GnRH and pituitary GnRHR (Figure 5), which were well consistent with the alterations of serum gonadotrophins (Table 3), delayed reproductive organ development (Table 2), and the manifestations of delayed puberty onset (vaginal opening and estrous cycle) (Figure 1). There seems to be a discrepancy between the stimulated GnRH secretion *in vitro* reported by Liu *et al.* [22] and the decreased hypothalamic GnRH mRNA expression and delayed vaginal opening (a marker of puberty) *in vivo* in our present study. To our opinion, this discrepancy may be attributed to several factors. On the one hand, T-2 toxin had been reported to possess a bi-directional modulatory activity. In a study of cultured human lymphocytes, enhanced immunoglobulin production was observed in cells exposed to lower doses of T-2 toxin, whereas decreased immunoglobulin production was noted at higher doses [32]. A different modulatory effect of T-2 toxin on the secretion of steroid hormone in granulosa cells was also observed. Maruniakova *et al.* [33] found that high dose of T-2 (1000 ng/mL) combined with 10 ng/mL insulin-like growth factor I (IGF-I) significantly stimulated progesterone release in porcine ovarian granulosa cells. On the contrary, a significant inhibition of progesterone release by low dose of T-2 toxin (100 ng/mL) combined with 10 ng/mL IGF-I was observed. In the study of Liu *et al.*, the stimulated GnRH secretion in GT1-7 cells *in vitro* was only observed in a relative low concentration range with the maximal effective concentration of 0.5 ng/mL. Considering the bi-directional modulatory activity of T-2 toxin mentioned above, we think the probability that higher doses of T-2 toxin exerts inhibitory activity on GnRH secretion in GT1-7 cells can not be excluded. And this hypothesis will also be tested in our future *in vitro* studies. Furthermore, T-2 toxin had been revealed to cause the impairment of the blood-brain barrier function [34], and to increase the blood-brain barrier permeability [35], both probably promote the entry of T-2 toxin into brain regions especially hypothalamus, resulting in the influence on HPG axis function. However, the metabolism and distribution of T-2 toxin in the brain following oral exposure are really unknown. Therefore, the concentration of T-2 toxin or its metabolites in different brain regions especially hypothalamus is unpredictable. Accordingly, it is hard to correlate the *in vitro* concentrations that stimulate GnRH secretion in GT1-7 cells with the *in vivo* exposure dose that delays puberty onset in female rats. On the other hand, unlike the regulation of GnRH secretion in cell cultures *in vitro*, the GnRH secretion and puberty onset *in vivo* are regulated by a complex network. The onset of puberty derives from the complex interplay among neuropeptides, neurotransmitters, and neurosteroids that occurs in the awakening of HPG axis [36]. It had been revealed that acute or repeated exposure to T-2 toxin could induce changes in the levels of brain neurotransmitters such as serotonin, dopamine and norepinephrine, which play important modulatory role in the onset of puberty [37,38,39]. Therefore, this difference between *in vitro* and *in vivo* regulatory mechanisms may at least in part explain the discrepancy between the observed *in vitro* and *in vivo* effects. Taken these points together, the delayed puberty onset in female rats induced by prepubertal exposure to T-2 toxin might be ascribed to its regulatory activity on hypothalamic function.

It is well confirmed that GnRH neurons play a pivotal role in regulating pubertal development and reproduction which are triggered by GnRH pulsatile secretion. The synthesis and secretion of GnRH is regulated by a complex and systematic network in hypothalamus, and kisspeptins the product of KiSS-1 gene and their G protein-coupled receptor 54 (GPR54) are recognized an essential gatekeeper in control of GnRH secretion and reproductive function [40,41,42]. In recent years, increasing studies have revealed the key role of kisspeptins in xenobiotics especially chemicals with endocrine disruption activity induced adverse effects on puberty onset and reproductive function [43,44,45,46]. As described above, our team recently finds that T-2 toxin has regulatory activity on GnRH secretion in hypothalamic GT1-7 cells. The further study reveals that exogenous kisspeptin-10 pretreatment elevates the reactivity of GT1-7 cells to T-2 toxin in light of GnRH secretion [22]. In that study, in the presence of 10 nM kisspeptin-10 pretreatment for 1 h that shows maximal GnRH release, the GnRH levels in GT1-7 cells were further increased after 3 h and 6 h of treatment with different concentrations of T-2 toxin. In addition, the change trends of protein expression of kisspeptins ligand GPR54 following T-2 toxin treatment, were consistent with GnRH changes in the absence or presence of exogenous kisspeptin-10. As discussed above, although there exists a discrepancy between the *in vitro* stimulatory effect on GnRH secretion and the reduced GnRH mRNA expression and delayed puberty onset *in vivo*, these *in vitro* findings provide a clue for us that T-2 toxin may disturb puberty onset in immature female rats by the perturbation of kisspeptins/GPR54 signaling pathway. Other central regulatory mechanisms such as the alterations of important neurotransmitters in brain should also be considered and investigated.

## 4. Experimental Section

### 4.1. Animals *(*Study Number 2011-005*)*

Healthy Sprague-Dawley (SD) rats were purchased from Laboratory Animal Center of Academy of Military Medical Sciences (Beijing, China), male rats weighed 300–350 g, and non-pregnant female rats were 250–300 g. The animals were maintained in a room with controlled illumination (12 h light/dark cycle), temperature (20 °C–25 °C), and humidity (40%–70%), and were given free access to regular rat diet and water. All the procedures were carried out according to the guidelines provided by the Animal Care and Facilities Committee, Institute of Disease Control and Prevention, Academy of Military Medical Sciences.

### 4.2. Chemicals and Reagents

T-2 toxin was kindly provided by the Institute of Pharmacology and Toxicology, Academy of Military Medical Sciences (Beijing, China). T-2 solutions were prepared in corn oil at concentration of 75.0 μg/mL. Rat luteinizing hormone (LH), follicle-stimulating hormone (FSH) and estradiol ELISA kits were obtained from RapidBio Lab (RapidBio, Hercules, CA, USA). Trizol reagent was obtained from Invitrogen Life Technologies Corporation (Carlsbad, CA, USA). cDNA synthesis kit was obtained from Fermentas International Inc (Burlington, ON, Canada). All the other chemicals were of analytical grade and were commercially available.

### 4.3. Experimental Procedures

#### 4.3.1. Mating and Experimental Design

The females were mated (two females per male), and the pregnant rats were housed individually. After birth, 10 female offspring were selected and raised by their consanguineous mother. If the number of female offspring was less than 10, it would be complemented by male ones. The female offspring were randomly assigned to two groups of 20 animals each. The dosing days in immature female rats in the present study were designed essentially in accordance with the OECD guideline 440 of Uterotrophic Bioassay in Rodents [47]. From postnatal day (PND) 15 to 19 (PND15-PND19), one group received a daily intragastric administration of T-2 toxin at a dose of 187.5 μg/kg body weight, and the other group received corn oil only (vehicle control). The dosing volume of T-2 toxin solution or corn oil was 2.5 mL/kg. Daily dosing was performed regularly in the morning from 8:30 A.M. to 9:00 A.M. The animals were weaned on PND21, and then were examined daily for vaginal opening and vaginal smears afterwards.

The single dose used in the present study was chosen mainly on the basis of the provisional maximum tolerable daily intake (PMTDI) of 60 ng/kg bw/day proposed by the WHO/FAO Joint Expert Committee on Food Additives (JECFA) for T-2 toxin [48], and the tolerable daily intake (TDI) of 100 ng/kg bw/day established by the European Food Safety Authority (EFSA) for the sum of T-2 and HT-2 toxins [49]. The dose chosen was briefly justified as follows. Classically, a PMTDI or TDI for a food additive or contaminant is allocated based on the most critical no observed adverse effect level (NOAEL) established in animal toxicological studies by applying an uncertainty factor of 100 or higher [50]. In the present study, we used a reverse deduction method, a NOAEL or “tolerable daily exposure dose” for T-2 toxin in rats was deduced from the existing PMTDI and TDI for humans by applying a higher uncertainty factor of 1000, considering the extrapolation differences (such as extrapolation from animal to human, extrapolation from high to low intake levels) and inter-individual variability. Accordingly, a NOAEL or “tolerable daily exposure dose” for T-2 toxin in rats was estimated in the range of 60–100 μg/kg bw/day. We, thus, chose a slightly higher dosage of 187.5 μg/kg bw/day, which exerts no significant adverse effects on the general behavior and growth of immature female rats in a preliminary test.

#### 4.3.2. Experiment I

In experiment I, 10 rats of each group were used to observe the days of vaginal opening (VO), first dioestrus (D1), and first estrus (E1) in regular estrous cycles. Vaginal smears were checked at the same time every day (8:00 A.M.–8:30 A.M.) from the day of vaginal opening until consecutive regular four- or five-day estrous cycles were established in all rats. On the day of consecutive regular estrous cycles were observed in the last rat, all rats were subject to euthanasia by cervical dislocation after deep anesthesia, and followed by autopsy. The thymus, liver, heart, kidney, spleen, uterus, ovary, bone, and other tissues/organs were harvested. The samples were fixed in 10% buffered formalin for 24 h at room temperature, embedded in paraffin, sectioned at 4 μm, stained with hematoxylin and feosin (H&E), and examined by light microscopy.

#### 4.3.3. Experiment II

In experiment II, the remaining 10 rats were used. Once vaginal opening was detected in rats of vehicle control group, these control rats were subject to euthanasia after deep anesthesia with pentobarbital (50 mg/kg bw) at 9:30 A.M. on the day. As the matches, the same number of T-2 toxin treated rats were randomly selected and sacrificed as well. Then all the rats were dissected in the morning (9:30 A.M.–11:00 A.M.) for histopathological examinations.

The blood was collected from the femoral artery before euthanasia, then was centrifuged at 3500× *g* for 15 min to obtain the serum. To avoid the possible differences in hormone levels due to diurnal rhythm, all blood collections were completed within 1 h. The resulting serum was frozen at −80 °C until hormone determination. Uterus, ovaries and vagina were dissected out of the surrounding fats and weighed, and their relative weights were calculated. The incidence of corpora lutea was calculated with stereomicroscope (Olympus, Tokyo, Japan). Then ovaries, uterus and vagina were fixed in 10% buffered formalin for the histopathological examination. The brain was immediately removed and the hypothalamus (limited anteriorly by the optic chiasma, laterally by the hypothalamic fissures, posteriorly by the mammilary bodies, and in depth by the subthalamic sulcus) and pituitary glands were dissected. Tissue samples were snap-frozen in liquid nitrogen, then were stored at −80 °C for the determinations of mRNA expressions of gonadotropin-releasing hormone (GnRH) and GnRH receptor (GnRHR).

### 4.4. Examination and Biochemical Determinations

#### 4.4.1. Vaginal Opening Examination

Daily vaginal opening (VO) examination was performed at 8:00 A.M. starting from PND20 by using surgical loupes. The day of VO was recorded as the day on which the vaginal orifice transitioned from tightly closed to patent [51].

#### 4.4.2. Hormone Assays

Serum levels of luteinizing hormone (LH), follicle stimulating hormone (FSH) and estradiol were measured by ELISA-methods (Rat LH, FSH and estradiol ELISA kits, RapidBio Lab, Hercules, CA, USA). The sensitivity limits were 0.3 ng/mL (LH), 1 ng/mL (FSH) and 5 pg/mL (estradiol), and results were expressed as ng/mL, ng/mL, and pg/mL, respectively.

#### 4.4.3. RNA Extraction and RT-PCR

Total RNA was extracted using Trizol reagent (Invitrogen, Carlsbad, CA, USA) according to the manufacturer’s instructions. cDNA synthesis and the analyses of the results obtained were performed as previously described [52]. Before analytical procedures, the purity and integrity of the RNA were examined spectroscopically (USA Technologies Inc, Malvern, PA, USA) and by gel electrophoresis (BioRad, Hercules, CA, USA). RNA samples (1 μg) were reverse transcribed adding Oligo d (T) (MBI Fermentas) and nuclease-free water in a volume of 12 μL, for 5 min at 65 °C. Reaction buffer, RNAase inhibitor, dNTP and MMLV reverse transcriptase were subsequently added and incubated for 60 min at 42 °C, then heated denatured for 5 min at 70 °C and stored at −20 °C. PCR primers and annealing temperature were as follows (Table 4). All primers were synthesized by Invitrogen^TM^ (Beijing, China). PCR cycling conditions were as follows: denaturation at 95 °C for 10 min, 40 cycles of denaturation at 95 °C for 30 s, annealing at 56 °C for 30 s and extension at 72 °C for 30 s. Twenty microliters of each PCR product was electrophoresed on a 1.5% agarose gel (with ethidium bromide) for 20 min at 120 V, then photographed (BioRad, Hercules, CA, USA). Densitometry was performed using Quantity One 6.0 software (BioRad, Hercules, CA, USA). The data were recorded as the ratio of the sample to the internal standard β-actin.

**Table 4:** PCR primers and annealing temperatures.

Gene Name	Sequence		Product Size (bp)	Annealing Temperature (°C)
GnRH	Sense	5′-AGCACTGGTCCTATGGGT TG-3′	105	56
	Antisence	5′-GGGGTTCTGCCATTTGATCCA-3′		
GnRHR	Sense	5′-GTATGCTGGAGAGTACTCTGCA-3′	380	56
	Antisence	5′-GGATGATGAAGAGGCAGCTGAAG-3′		
β-actin	Sense	5′-TCGGTCATCACTATCGGCAAT-3′	550	56
	Antisence	5′-GTATGCTGGAGAGTTACTCTGCA-3′		

### 4.5. Statistical Analysis

The differences between vehicle control and toxin-treated group were analyzed by *t* test using SPSS 16.0 statistical software (SPSS, Chicago, IL, USA), with the significance level set at *p* < 0.05 or *p* < 0.01. All data are expressed as mean ± SD.

## 5. Conclusions

Our present results clearly reveal that prepubertal exposure to T-2 toxin delays the onset of puberty in female rats. However, the key point that remains to be elucidated in future studies is whether the postponed puberty onset induced by T-2 toxin is mainly attributed to the disturbance of hypothalamic function, particularly the kisspeptins/GPR54 signaling pathway. Additionally, the dose-response and the BMDL or NOAEL for the perturbation activity of T-2 toxin on puberty onset in immature female rats still need illumination. Nevertheless, since there is few epidemiologic or experimental data on the adverse effect of T-2 toxin on the onset of puberty in females, this study provides new information for the reproductive and developmental toxicity of T-2 toxin. In recent years, special emphasis has been placed on the evaluation of dietary intake of T-2 toxin in high-risk sub-groups of the population, especially children. The EFSA estimated the total chronic dietary exposure to T-2 plus HT-2 toxins in toddlers across 14 European countries to be between 12 and 43 ng/kg bw/day for mean consumers and between 23 and 91 ng/kg bw/day for 95th percentile consumers [49]. It has also been reported that exposure to T-2 plus HT-2 toxins in children from France and Catalonia (Spain) is relative high, and occasionally exceeds the TDI of 100 ng/kg bw/day [53,54]. As we have justified, the dose of T-2 toxin used in the present study is a “tolerable daily exposure dose” deduced from the PMTDI and TDI for human exposure. Therefore, the observed disturbance of puberty onset in immature female rats, which was induced by short-term prepubertal exposure to a “tolerable daily exposure dose” of T-2 toxin, has a realistic significance. Considering the vulnerability of developmental children to food contaminants and the relative high level of dietary intake of T-2 toxin in children, we think the findings of our present study may provide valuable information for the health risk assessment in children.
